# Treatment of distal clavicle fractures using a Scorpion plate and influence of timing on surgical outcomes: a retrospective cohort study of 105 cases

**DOI:** 10.1186/s12891-020-3169-9

**Published:** 2020-03-04

**Authors:** Ryogo Furuhata, Masaaki Takahashi, Teppei Hayashi, Miyu Inagawa, Aki Kono, Noboru Matsumura, Yusaku Kamata, Hiroshi Arino, Hideo Morioka

**Affiliations:** 1 Department of Orthopaedic Surgery, National Hospital Organization Tokyo Medical Center, 2-5-1, Higashigaoka, Meguro-ku, Tokyo, 152-8902 Japan; 2 Department of Orthopaedic Surgery, Keio University School of Medicine, Shinjuku-ku, Tokyo, Japan

**Keywords:** Distal clavicle fracture, Plate, Scorpion plate, Outcome, Complication, Union, Timing of surgery

## Abstract

**Background:**

Plate fixation is an established method for treating unstable distal clavicle fractures. However, the appropriate timing of surgery for acute distal clavicle fractures remains unclear. The present study aimed to evaluate the clinical outcomes of osteosynthesis using a Scorpion plate and to assess the influence of surgery timing on the surgical outcomes for acute unstable distal clavicle fractures.

**Methods:**

We retrospectively reviewed 105 patients who underwent fixation for acute unstable distal clavicle fractures (Neer type II and V) using the Scorpion plate between 2008 and 2018. Patients were divided into early (45 patients) and delayed (60 patients) treatment groups based on the timing of the surgical intervention (within or after 7 days). The outcomes were postoperative complications (nonunion, peri-implant fracture, plate loosening, plate-related pain, and stiffness). We evaluated the outcomes from X-ray radiographs and clinical notes.

**Results:**

Among the 105 patients, nonunion, plate loosening, plate-related pain, and stiffness were observed in six patients (5.7%), four patients (3.8%), seven patients (6.7%), and one patient (1.0%), respectively. The nonunion rate was significantly higher in the delayed treatment group than that in the early treatment group (*P* = 0.036). Although the difference was not significant, plate loosening and stiffness were only observed in the delayed treatment group.

**Conclusion:**

Our results demonstrated that osteosynthesis using Scorpion plates achieved satisfactory surgical outcomes for unstable distal clavicle fractures. In addition, this study suggested that performing surgery within 6 days after injury is recommended to reduce postoperative complications.

## Introduction

**Fig. 1:**
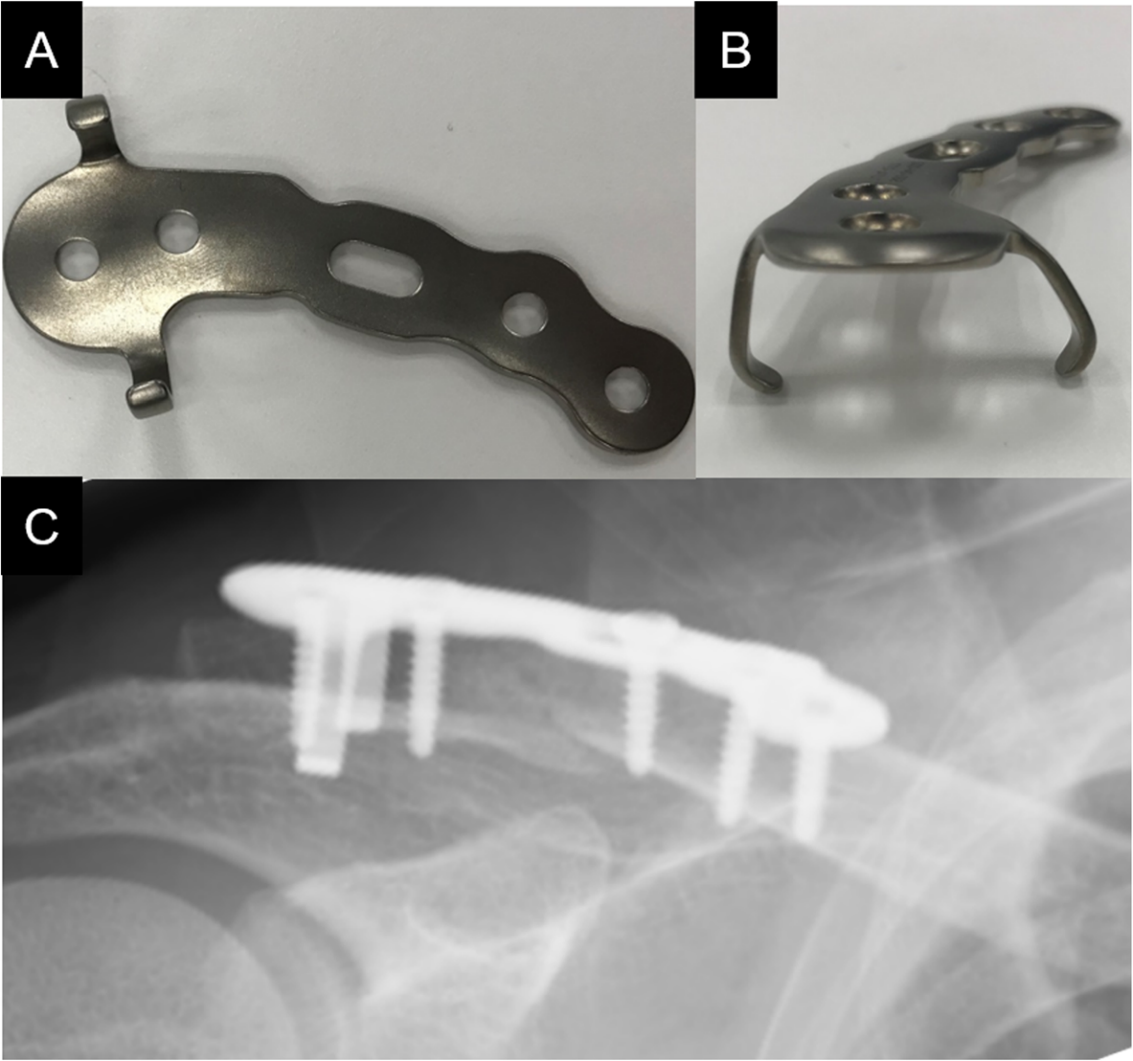
The photo images demonstrate a SCORPION® plate (**a** and **b**). Plain radiograph showing osteosynthesis using a SCORPION® plate within six days after injury (**c**)

**Fig. 2:**
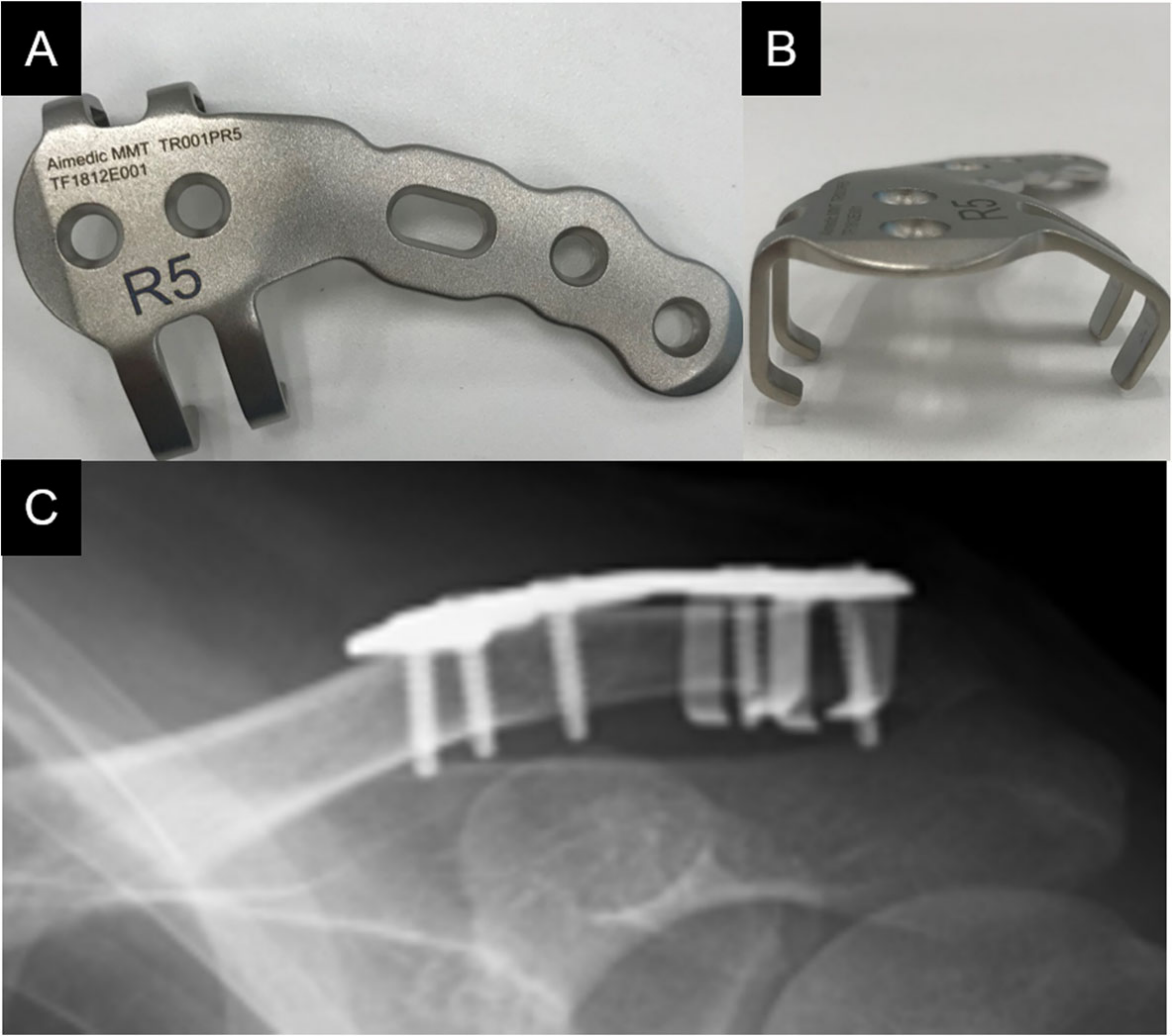
The photo images demonstrate a SCORPION® NEO plate (**a** and **b**). Plain radiograph showing osteosynthesis using a SCORPION® NEO plate within six days after injury (**c**)

Distal clavicle fractures have a high nonunion risk (22–50%) in patients treated non-surgically due to the instability caused by the two opposing forces of trapezius traction and the weight of the upper extremity [1–3]. Therefore, for unstable distal clavicle fractures classified as Neer type II or V [4, 5], surgical treatment is usually indicated [6]. To date, we have used the anatomical non-locking plates, “SCORPION®” (Fig. 1a and b) and “SCORPION® NEO” (Fig. 2a and b) (Aimedic MMT, Tokyo, Japan), to treat unstable distal clavicle fractures. In fixation with Scorpion plate, the distal bone fragment is grasped by the plate arm and fixed with one or two screws, while the proximal bone fragment is fixed with three screws. The bending stiffness and torsional stiffness of the Scorpion plate were reported to be significantly higher than those of tension band wiring and were equivalent to those of hook plates [7]. However, there have been no large clinical studies on the clinical results of osteosynthesis using Scorpion plates.

Plate fixation for distal clavicular fractures has been reported to achieve satisfactory bone union rates [8–14]; however, one of the important clinical problems is the rate of postoperative complications. While hook plates showed good outcomes for fractures with comminuted distal bone fragments, various complications, such as peri-implant fracture, dislocation, and limited range of motion were reported [6, 10, 15]. On the other hand, plates that are fixed proximal to the acromioclavicular joint have been reported as leading to fewer complications than the hook plate, since the plates do not disturb the acromioclavicular joint motion [14, 16, 17]. However, complications such as plate fracture due to trauma, nonunion, and plate loosening can occur, albeit at a lower frequency, and some cases require re-operation [18, 19]. Nevertheless, risk factors for these postoperative complications remain largely unknown. The incidence of postoperative complications is reported to be high in patients for whom more than 4 weeks had elapsed since the injury [16, 20]. These reports suggest that the time to surgery may affect the occurrence of postoperative complications. However, the appropriate timing of surgery for acute distal clavicle fractures is still unclear. We hypothesized that delayed surgical intervention would make the anatomical reduction difficult and lead to higher complications than early intervention.

The purpose of this study was to evaluate the clinical outcomes of distal clavicle fractures using the Scorpion plate and to assess the appropriate timing of surgery for acute unstable distal clavicle fractures.

## Methods

### Patient selection

This was a retrospective study of patients who underwent osteosynthesis using Scorpion plates for unstable distal clavicle fractures (Neer type II or V) between 2008 and 2018. During this period, SCORPION® plates were used from April 2008 to March 2014, and SCORPION® NEO plates were used from April 2014 to March 2018. The surgical techniques for these two plates are generally the same, with the exception of the presence of one plate arm in SCORPION® plates grasping the distal fragment compared with the two plate arms in SCORPION® NEO plates. The procedures were performed at a single general hospital by 14 surgeons (all trainees who were not specialized in shoulder surgery). We included patients with acute clavicle fractures within 3 weeks of injury. We excluded patients with inadequate follow-up, which was defined as the inability to continue follow-up until radiographically confirmed fracture union was observed or the cases in which the follow-up of patients was not feasible for at least 6 months after surgery.

We identified 110 patients who met the inclusion criteria. Five patients were excluded due to inadequate follow-up. The mean (± standard deviation) age of patients at the time of surgery was 47.0 ± 16.7 years (range: 14–89 years). The affected side was the right for 58 patients and the left for 47 patients. There were 55 smokers (52.3%). The type of clavicle fracture was Neer classification type IIA in 23 patients (22.0%), type IIB in 63 patients (60.0%), and type V in 19 patients (18.1%) [4, 5]. The type of plates used was SCORPION® plate (60 patients, accounting for 57.1%) and SCORPION NEO® plate (45 patients, accounting for 42.9%). Patients who met these criteria were further divided into two groups; an early treatment group (early group), which included patients for whom the time from injury to surgery was within 6 days, and a delayed treatment group (delayed group), which included patients for whom time to surgery was 7 days or more. This cut-off was set based on a previous report on acute proximal humeral fracture for which delayed surgical intervention (six or more days after the injury) was related to a significant increase in postoperative complications [21] because there was no reference describing the timing of surgery for acute distal clavicular fractures. The early group included 45 patients and the delayed group included 60 patients (Table 1).

**Table 1:** Patient demographics

	Early group (*n* = 45)	Delayed group (*n* = 60)	*P* value
Time from injury to surgery (days)	3.9 ± 1.5	10.7 ± 3.4	< 0.001 *
Age (years)	44.4 ± 14.9	49.0 ± 17.6	0.194
Male/Female	35/10	50/10	0.473
Side of Injury, Right/Left	27/18	31/29	0.395
Smoker/Non-smoker	26/19	29/31	0.338
Neer Type IIa	7	16	0.173
Neer Type IIb	30	33	0.227
Neer Type V	8	11	0.942
Type of plate, SCORPION®/SCORPION NEO®	23/22	37/23	0.279
Additional fixation with Kirschner wire or suture anchor	7	13	0.430

* *P* < 0.05

### Surgical procedure

**Fig. 3:**
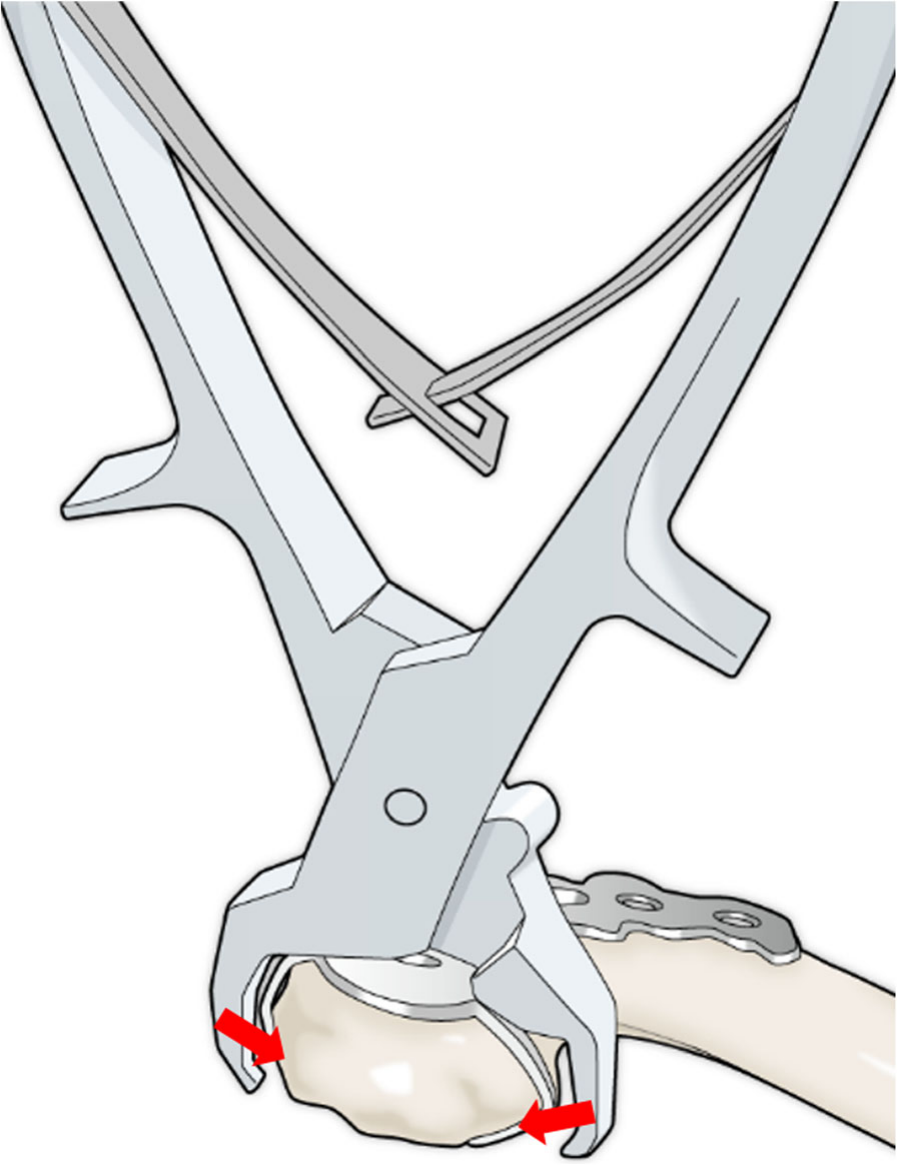
A schematic image showing that the plate arm was bent using a specialized instrument so that it firmly grasped the clavicle

**Fig. 4:**
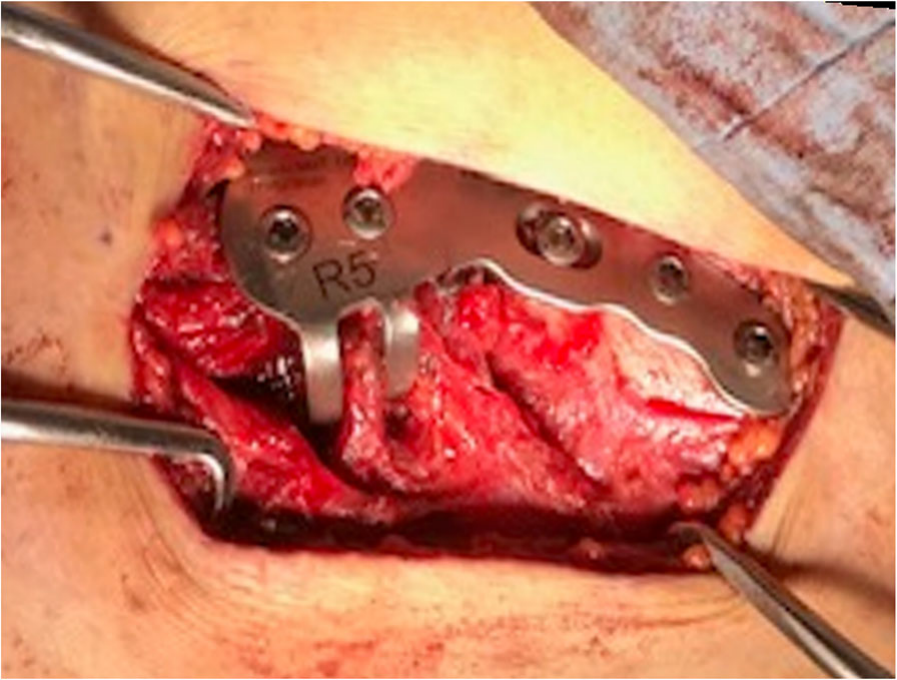
Intraoperative image after osteosynthesis with the Scorpion plate. In the osteosynthesis using a SCORPION® NEO plate, two anteroposteriorly folded plate arms compressed and grasped the distal fragment. Two distal and three proximal cortical screws were then inserted to fix the plate

All patients underwent fracture fixation in a beach chair position under general anesthesia. The approach was from the superior side of the clavicle, and the acromioclavicular joint capsule was preserved. After temporarily fixing distal bone fragments with 2.0 mm Kirschner wires under fluoroscopy, if any third bone fragments were present, we sutured these fragments to the clavicle using high-strength absorption sutures. We ultimately fixed these fracture fragments using a SCORPION® plate (Fig. 1c) or a SCORPION® NEO plate (Fig. 2c). Two distal and three proximal cortical screws were inserted to fix the plates. These plates are characterized by one plate arm attached to SCORPION® plates and two plate arms in SCORPION NEO® plates. In addition, these plate arms are bent with a specialized instrument to pressure-fix the distal fragment (Figs. 3 and 4). If the fixation was considered to be insufficient, fixation with 2.0 mm Kirschner wire, or coracoclavicular ligament reconstruction with a suture-anchor, was additionally performed. After surgery, the affected arm was kept in a sling for 1–3 weeks depending on the postsurgical fixation strength. Pendulum exercises were started the day after surgery and passive and active range of motion exercises were permitted after 1 week. Implant removal was performed when bone union was obtained at least 6 months after the operation and in the case that the patient requested the removal.

### Outcomes

The outcomes were the occurrence of complications (nonunion, peri-implant fracture, plate loosening, plate-related pain, and stiffness requiring arthroscopic capsulotomy) and postoperative fracture gap. A single examiner, who did not participate in any surgeries, blindly evaluated the plain radiograph images; thereafter, evaluation of the clinical outcomes and assignment of the early and delayed groups were performed by clinical notes to reduce the bias of measurements. According to a previous report [22], nonunion was defined as a lack of bone bridging after more than 12 months after injury. We defined plate loosening as any screws backing out or a plate displacement greater than 3 mm above the clavicle. Pain from the plate fixation was evaluated based on patient complaints 6 months after surgery (prior to implant removal). We compared the incidence of postoperative complications between the early group and delayed group.

To further evaluate whether there were any differences in outcomes between the SCORPION® plates and SCORPION NEO® plates, we compared the incidence of postoperative complications between the groups using SCORPION® plate and SCORPION NEO® plate.

### Statistical analysis

All statistical analyses were conducted using SPSS version 26.0.0.0 (IBM, Armonk, NY). We used Student’s *t*-tests to compare the average of continuous values (age, time from injury to surgery, and postoperative fracture gap) and chi-square tests to compare the proportion of discrete variables (sex, the side of injury, smoking, type of fracture, type of the plates, and additional fixation with Kirschner wire or suture anchor) between the early and delayed groups. When comparing the proportion of variables, especially if the expected value of the variable was less than five (nonunion, plate loosening, peri-implant fracture, plate-related pain, and stiffness requiring arthroscopic capsulotomy), Fischer’s exact test was used. Continuous data are presented as mean ± standard deviations (SD). The threshold for significance was *P* < 0.05.

## Results

In this study, although there was a significant difference in the time from injury to surgery between the early and delayed groups (*P* < 0.001), there were no significant differences in age, sex, affected side, smoking rate, type of clavicle fracture, type of plates and additional fixation with a Kirschner Wire or a suture-anchor (Table 1). In addition, 28 patients from the early group and 40 patients from the delayed group removed the implants, which showed no significant difference between the early and delayed groups.

Among the 105 patients, nonunion 1year after surgery and plate loosening were observed in six (5.7%) and four patients (3.8%), respectively. Nonunion was only observed in the delayed group, and the frequency of nonunion was significantly higher in the delayed group than that in the early group (*P* = 0.036). Plate loosening was also only observed in the delayed group, but no significant difference was observed in comparison with the early group. Peri-implant fractures were not observed in either group. There was no significant difference in the frequency of plate-related pain at 6 months after surgery between the two groups. Postoperative shoulder stiffness was observed in one patient in the delayed group, which required arthroscopic capsulotomy 2years after surgery. In addition, the postoperative fracture gap was significantly greater in the delayed group; it was 0.9 ± 1.7 mm in the early group and 2.0 ± 2.0 mm in the delayed group (*P* = 0.004) (Table 2).

**Table 2:** Comparison of incidence of postoperative complications and postoperative fracture gap (early group vs. delayed group)

	Early group (n = 45)	Delayed group (n = 60)	P value
Nonunion (one year after surgery)	0 (0%)	6 (10%)	0.036*
Plate loosening	0 (0%)	4 (6.7%)	0.133
Peri-implant fracture	0 (0%)	0 (0%)	–
Plate-related pain	3 (6.7%)	4 (6.7%)	1
Stiffness requiring arthroscopic capsulotomy	0 (0%)	1 (1.7%)	1
Postoperative fracture gap (mm)	0.9 ± 1.7	2.0 ± 2.0	0.004*

* *P* < 0.05

**Fig. 5:**
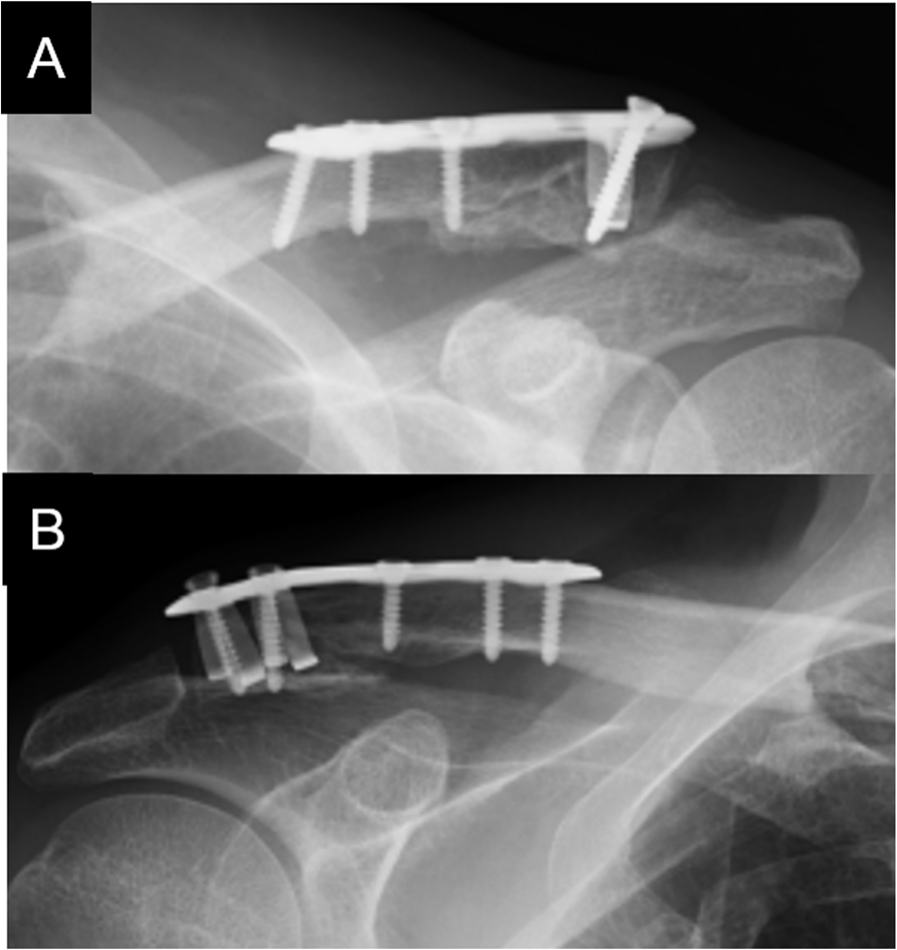
Plain radiographs showing delayed bone union. Post-operative radiographs performed 1 year after surgery revealed that the fracture line is visible and the distal screws were loosening (**a** and **b**)

Among the six patients with nonunion, only one patient experienced shoulder pain during elevation and a mild limitation of the range of motion, while other patients were asymptomatic. In addition, a loosening of the screw inserted into the distal bone fragment was observed on radiographic images in two patients (Fig. 5). All patients with nonunion at 1 year were observed nonoperatively and bone union was finally obtained within 1.5 years (Table 3).

**Table 3:** Details of patients experiencing delayed bone union. (0 females, 6 males, age range 24–72 years, median 38.5 years)

	Age /Sex	Type (Neer)	Symptoms	Imaging findings	Clinical results
Patient 1	32 /male	IIA	Asymptomatic	Residual postoperative fracture gap (> 3 mm)	Bone union at 1.2 years
Patient 2	24 /male	IIB	Asymptomatic	–	Bone union at 1.3 years
Patient 3	34 /male	IIB	Mild limitation in elevation (135°) Pain at elevation	Distal screw loosening	Bone union at 1.5 years
Patient 4	72 /male	V	Asymptomatic	–	Bone union at 1.3 years
Patient 5	57 /male	IIB	Asymptomatic	Distal screw loosening	Bone union at 1.5 years
Patient 6	43 /male	V	Asymptomatic	–	Bone union at 1.3 years

Additionally, we compared the outcome between the groups using SCORPION® plate and using SCORPION NEO® plate. The proportion of males and additional fixation with Kirschner wire or suture anchor in the SCORPION NEO® plate group was smaller than the proportion obtain in the SCORPION® plate group (*P* < 0.05); however, there were no significant differences in the time from injury to surgery, age, affected side, smoking rate, and type of clavicle fracture (Supplementary Table 1). There were also no significant differences in the incidence of postoperative complications between the SCORPION® plate and SCORPION NEO® plate groups, although the incidence of plate loosening tended to be lower in the group using SCORPION NEO® plates (0% vs 6.7%, *P* = 0.133) (Supplementary Table 2).

## Discussion

In this study, we made two important clinical observations. First, results of this study indicated that osteosynthesis using Scorpion plates constitute a promising surgical treatment for unstable distal clavicle fractures. Second, this study suggested that delaying the surgical intervention for more than 6 days after injury is associated with the increased occurrence of nonunion 1 year after surgery in osteosynthesis using Scorpion plates for distal clavicle fractures.

First, our present results demonstrated that osteosynthesis using Scorpion plates for unstable distal clavicle fractures led to high bone union and low complication rates. In this study, six patients (5.7%) experienced nonunion 1 year after surgery and one patient was symptomatic. However, bone union was ultimately achieved in all patients within 1.5 years after surgery. Previous studies investigating the clinical outcomes of at least 30 patients with distal clavicle fractures showed a bone union rate of 94–100% with hook plates [10, 12, 14] and 97–100% with plates not fixed across the acromioclavicular joint [13, 14]. Our findings suggest that the postoperative bone union rate observed with Scorpion plates can be equivalent to that of other plates. In distal clavicle fractures, distal fragments are fragile and often comminuted; therefore, stable fixation with screws is not always obtained. Scorpion plates can be used to fix distal fragments in a manner such that their arms wrap them with soft tissue en bloc. However, plate loosening was observed in four patients (3.8%), presumably because Scorpion plates do not have locking screws. In all these patients, the loosening of screws inserted into distal bone fragments was observed, and the length of the distal screw was considered insufficient. These observations indicate that when performing fixation with the Scorpion plate, it is crucial to select the optimal distal screw length since distal bone fragments were fixed with only one or two screws in addition to the plate arm. Additionally, in this study, there were no reports of plate loosening in the SCORPION NEO® group, whereas plate loosening was observed in four patients (6.7%) in the SCORPION® plate group. Although significant differences were not observed, there is the possibility that the number of plate arms might affect the bone fragment grasping force of the plates.

Second, this study demonstrated that the rate of nonunion was significantly higher when osteosynthesis using Scorpion plates was delayed for 7 days or more after injury. In addition, although the difference in frequency was not significant, plate loosening and stiffness were only seen in the delayed surgery group. These results suggest that delaying the surgical intervention for seven or more days after injury may be associated with an increase in postoperative complications of osteosyntheis using Scorpion plates for acute distal clavicle fractures. Regarding the timing of distal clavicle fracture surgery, the timing of surgery (less than 10 weeks after the injury vs. at least 10 weeks thereafter) reportedly did not affect clinical outcomes [23], while postoperative complications have been reported to increase in subjects that undergo surgery more than 4 weeks after injury [16, 20]. However, the timing of surgery for acute fractures remains unclear. Recently, proximal humerus fracture fixation within 5 days of the fracture event was recommended, since a delayed intervention (six or more days after the injury) is related to a significant increase in complications [21], which is similar to the results of this study. A delayed surgical intervention is thought to complicate the anatomical fracture reduction and increase soft tissue dissection, which may result in a longer fracture union time [24]. In this study, the postoperative fracture gap is significantly greater in the delayed group, which supports the hypothesis that delayed surgical intervention makes the anatomical reduction difficult [24]; furthermore, it can explain our finding of a higher nonunion rate in the delayed surgery group compared with the early group. In this study, one patient who experienced nonunion 1 year after the operation was symptomatic (16.6%), but reoperation was not required, and bone union was finally obtained 1.5 years after surgery. However, considering that some cases of nonunion or plate loosening associated with delayed union were reported to require reoperation with iliac bone grafting [18, 19], performing osteosynthesis using Scorpion plates for acute distal clavicle fractures within 6 days after the injury would be preferable.

The incidence of complications after plate fixation for distal clavicular fractures has been reported to be 22% in a large-scale systematic review [14]; therefore, based on a power analysis assuming a 20% rate of postoperative complications, approximately 400 patients in total would be required in this study to show a 50% difference in the risk of postoperative complications. Based on this, the study may include effects owing to the timing of surgery and type of plates that could not be detected (β-error). However, the number of fractures were relatively large in this clinical study on more than 100 patients over 10 years in this study, while a majority of previous studies on the surgical outcomes for distal clavicle fractures had a sample size of 50 or less. This can be considered a major strength of this study. In addition, we used Scorpion plates for all unstable distal clavicle fractures and the surgical procedure was standardized during the period, suggesting that the generalization of clinical results using Scorpion plates is possible.

In contrast, there were several major limitations to this study. First, it is difficult to accurately evaluate the superiority or inferiority of Scorpion plate to other implants since this study was not a comparative study between different implants. Second, because of this study’s observational design, biases from unobserved differences may have affected the results. For example, the procedures were performed by 14 surgeons; however, the influence of the abilities of the surgeons or the assistants were not evaluated. Although smoking was reported as a risk factor for nonunion of distal clavicle fractures [19], smoking status was adjusted between the early and delayed groups in this study. Third, the state of the acromioclavicular joint could be another possible limitation. Because the coracoclavicular ligament can be damaged with distal clavicle fracture, acromioclavicular joint separation might concur following osteosynthesis. However, we could not accurately evaluate the state of the acromioclavicular joint because most of our cases lacked the radiographs of the contralateral acromioclavicular joint. Finally, since questionnaire surveys were not administered in this study, it was not possible to determine additional objective functional outcomes.

## Conclusion

This study provides new information on the clinical effectiveness of Scorpion plates in the treatment of distal clavicle fractures. Further, as osteosynthesis using Scorpion plates for acute unstable distal clavicle fractures, performing surgery within 6 days after injuries is recommended to reduce postoperative complications.

## Supplementary information

**Additional file 1: Supplementary Table 1.** Patient demographics (Osteosynthesis using SCORPION® vs. SCORPION NEO®).

**Additional file 2: Supplementary Table 2.** Comparison of incidence of postoperative complications (Osteosynthesis using SCORPION® vs. SCORPION NEO®).

## Data Availability

Data that support the findings of this study are available from the corresponding author on reasonable request.

## Abbreviations

SD: Standard deviation
