# Supplementary material for: Treatment of distal clavicle fractures using a Scorpion plate and influence of timing on surgical outcomes: a retrospective cohort study of 105 cases
Source: BMC Musculoskelet Disord. 2020 Mar 4;21:146. doi: 10.1186/s12891-020-3169-9 (PMC7057610; doi:10.1186/s12891-020-3169-9)
Supplement: Supplementary file 1 — Additional file 1: Supplementary Table 1. Patient demographics (Osteosynthesis using SCORPION® vs. SCORPION NEO®). [file 12891_2020_3169_MOESM1_ESM.docx]

**Supplementary Table 1. Patient demographics (Osteosynthesis using SCORPION® vs. SCORPION NEO®)**

|  | SCORPION®  (n=60) | SCORPION NEO®  (n=45) | P value |
| --- | --- | --- | --- |
| Time from injury to surgery (days) | 8.2±4.2 | 7.6±4.4 | 0.478 |
| Age (years) | 46.4±16.1 | 48.1±17.1 | 0.597 |
| Male/Female | 54/6 | 31/14 | 0.006 * |
| Side of Injury, Right/Left | 38/22 | 20/25 | 0.054 |
| Smoker/Non-smoker | 32/28 | 23/22 | 0.821 |
| Neer Type IIa | 17 | 7 | 0.123 |
| Neer Type IIb | 32 | 30 | 0.169 |
| Neer Type V | 11 | 8 | 0.942 |
| Additional fixation with Kirschner wire or suture anchor | 16 | 4 | 0.022 * |

* P<0.05
