# Supplementary material for: Treatment of distal clavicle fractures using a Scorpion plate and influence of timing on surgical outcomes: a retrospective cohort study of 105 cases
Source: BMC Musculoskelet Disord. 2020 Mar 4;21:146. doi: 10.1186/s12891-020-3169-9 (PMC7057610; doi:10.1186/s12891-020-3169-9)
Supplement: Supplementary file 2 — Additional file 2: Supplementary Table 2. Comparison of incidence of postoperative complications (Osteosynthesis using SCORPION® vs. SCORPION NEO®). [file 12891_2020_3169_MOESM2_ESM.docx]

**Supplementary Table 2. Comparison of incidence of postoperative complications (Osteosynthesis using SCORPION® vs. SCORPION NEO®)**

|  | SCORPION®  (n=60) | SCORPION NEO®  (n=45) | P value |
| --- | --- | --- | --- |
| Delayed union | 4 (6.7%) | 2 (4.4%) | 0.698 |
| Plate loosening | 4 (6.7%) | 0 (0%) | 0.133 |
| Peri-implant fracture | 0 (0%) | 0 (0%) | - |
| Plate-related pain | 4 (6.7%) | 3 (6.7%) | 1 |
| Stiffness requiring arthroscopic capsulotomy | 0 (0%) | 1 (2.2%) | 1 |

* P<0.05
